# Awareness of COVID-19 Before and After Quarantine Based on Crowdsourced Data From Rabigh City, Saudi Arabia: A Cross-Sectional and Comparative Study

**DOI:** 10.3389/fpubh.2021.632024

**Published:** 2021-04-07

**Authors:** Mai Aldhahri, Rana Alghamdi

**Affiliations:** Department of Chemistry, Science and Arts College, King Abdulaziz University, Jeddah, Saudi Arabia

**Keywords:** COVID-19, SARS-CoV, Rabigh, quarantine, Saudi Arabia

## Abstract

**Background:** Infection prevention and control measures are critical for the prevention of the spread of COVID-19.

**Aim:** In this study, we aimed to measure and evaluate the level of awareness and knowledge of the prevention, symptoms, and transmission control of COVID-19 before and after quarantine among the residents of Rabigh city and adjacent villages in Saudi Arabia.

**Methods:** A cross-sectional online survey was conducted in two stages: the first stage took place before quarantine and the second stage took place after quarantine. The survey was filled out electronically.

**Results:** A total of 448 participants responded and filled out the questionnaires. Females (73.70%) formed the largest number of participants for both stages. The majority of the participants were <30 years old (50.90%) and had a high education level in various sectors and levels (97.1%). It was noticeable that during the first stage, the participants' awareness of COVID-19 symptoms was not very high: 13.62% did not know about the symptoms. However, by the second stage, awareness about symptoms had increased (9.6%).

**Conclusion:** The residents of Rabigh city and the surrounding villages had good levels of knowledge about COVID-19.

## Introduction

Over the last two decades, several different viral epidemics have occurred, such as severe acute respiratory syndrome coronavirus (SARS-CoV), the Middle East respiratory syndrome coronavirus (MERS-CoV), and severe acute respiratory syndrome coronavirus 2 (SARS-CoV-2) (1–3).

Coronaviruses are a group of related RNA viruses that cause diseases in mammals and birds and frequently lead to severe clinical symptoms and mortality in humans (4, 5). The native hosts of SARS-CoV, MERS-CoV, and SARS-CoV-2 are bats, who mostly transmit these viruses indirectly (6–8).

The first cases of novel SARS-CoV-2 (COVID-19) were recorded in December 2019 (1). Initially, coronavirus spread rapidly across China, Thailand, Japan, South Korea, and the USA; it then spread to almost 216 international locations (9–12). After the worldwide spread of COIVID-19 substantially increased, the International Health Regulations Emergency Committee of the World Health Organization announced the outbreak on 30 January 2020 (13).

COVID-19 is the second coronavirus to affect Saudi Arabia following MERS-CoV in 2012 (14), which was first reported in Saudi Arabia on 2 March 2010 in a Saudi citizen who had returned from Iran through Bahrain (15). To date, more than 49.7 million confirmed cases of COVID-19 infections have been identified globally, with more than 1.2 million confirmed deaths (as of 8 November 2020). Saudi Arabia has also been seriously affected by the COVID-19 pandemic, reporting its first confirmed case on 3 March 2020. The numbers increased dramatically and reached 350,229 on 8 November 2020, with 5,525 confirmed deaths across the kingdom (16). The first case in Rabigh city was detected on 14 April 2020, with 272 cases confirmed by 14 November 2020, the highest number of cases to date (17).

As cases escalated, starting from March 23, 2020, the Saudi Arabian government took precautions, such as implementing a compulsory quarantine with a lockdown curfew and social distancing to both protect public health and minimize negative political consequences (18). The compulsory quarantine and lockdown curfew lasted until 21 June 2020 (19). In Saudi Arabia, 89% of the population use the internet and 96% use smartphones, so during the COVID-19 pandemic, the Saudi government implemented applications and recommended them for digital disease containment measures to meet the community's needs and demands in a timely manner while maintaining quick and timely digital data sharing and follow up (20, 21).

All ages are susceptible to COVID-19, but a difference lies in the symptoms. Reports show that older patients, with an average age of 45, are more likely to catch the disease than younger patients, experience more severe symptoms and are at greater risk of death due to chronic conditions and biological differences (22). In mainland China, the delay in seeking healthcare or going to a hospital or clinic was initially on average 2 days, but as awareness of COVID-19 increased, this delay became shorter (22).

Most people infected with COVID-19 have symptoms, but some have slight or no symptoms (23). Fever is reported in 98% of confirmed cases and is the primary and basic sign of COVID-19 (1). After a fever, the most common symptoms reported at the onset of the illness are cough (76%), myalgia or fatigue (44%), dyspnea (55%) and sputum production (28%) (1). Another study reported the following common symptoms: dry cough (60–86%), shortness of breath (53–80%), and fatigue (38%) (24).

Effective actions can be taken to protect against COVID-19 infection and limit the spread of the virus. The first line of defense, to lower the risk of an epidemic, is a face mask and hand hygiene, which give on average 43% protection from the virus (25). Another study proved that a 6–44% uptake of hand hygiene could break the virus cycle and reduce the extent of the pandemic (26).

The fight against COVID-19 continues worldwide, and to guarantee success, people's adherence to preventive measures is essential. This study aimed to assess and evaluate the level of awareness and knowledge of COVID-19 prevention, symptoms, and transmission control before and after the compulsory quarantine of residents of Rabigh city and adjacent villages in Saudi Arabia.

## Methods

### Study Population

This cross-sectional study was conducted through an online survey in two stages: before and after the quarantine. The study participants were residents of Rabigh city and surrounding villages, the main selection criterion. A total of 448 participants responded and filled out the questionnaires.

The questionnaires were publicly available and the links to the online questionnaires were given to the participants through various social media platforms and emails. The participants electronically supplied their written informed consent to participate in the study before completing and submitting the questionnaires. The authors contacted the Science and Arts College, Rabigh Campus, for ethical approval; however, it confirmed that ethical approval was not required for this study in accordance with local legislation and national guidelines.

### Questionnaire and Data Collection

The research questionnaires were formulated based on the information given by the Saudi Ministry of Health for COVID-19, including common and specialized questions. Google Forms was used to write the questionnaires (the online links to the questionnaires are provided in the Supplementary Materials). The first questionnaire was distributed on 3 March 2020, and the second questionnaire was distributed on 6 October 2020, after the compulsory quarantine. The second questionnaire collected information about the participants' recall of their knowledge and attitude during the quarantine. The two questionnaires contained the same questions unless otherwise stated. Responses from participants who were not residents of Rabigh city or surrounding villages were not included in the study.

The questionnaires were divided into multiple sections. The first section collected demographic aspects of the study population, including personal data (age, sex, marital status, level of education, occupation and whether the participant was a resident of Rabigh city or its villages). The second section contained multiple-choice questions that asked if the participants were complying and adhering to the COVID-19 prevention regulations distributed by the Saudi Ministry of Health: wearing a face mask, keeping social distancing, the number of handwashes per day, and using the proper handwashing and hand-hygiene recommendations. The answers to questions in this section were either *yes, no* or *sometimes* or *less than 3, from 4 to 10* or *more than ten times* for the number of handwashes per day. The third section collected the participants' responses as either *yes, no* or *I don't know* to questions designed to test their knowledge of the main symptoms of COVID-19 and prevention methods. The fourth section included specific questions that explored the sources that the participants relied on for COVID-19 information, including magazines, TV, and social media. The last section of the questionnaire was only applicable to participants who responded after the quarantine. This section explored whether the participants were using any of the mobile phone applications (i.e., Tawakkalna, Tatamman, and Tabaud) that the Saudi government had recommended during the compulsory quarantine and lockdown (information about these phone applications can be found in the Supplementary Materials). A single-item scale was used to record the respondents' replies.

### Statistical Analysis

Data obtained from the questionnaires were compiled and statistically analyzed by Statistical Package for Social Science (SPSS, Version 23.0). The variables were displayed as frequencies and percentages. Multiple regression analyses for differences were considered to be statistically significant at *p* ≤ 0.05.

## Results

### Demographic Aspects of the Study Population

The total number of participants was 488: 159 filled out the questionnaire distributed before the quarantine and 253 filled out the questionnaire distributed after the quarantine. The respondents' demographics are summarized in Table 1. The respondents comprised 118 (26.30%) males and 330 (73.70%) females. About 50% (228) of the respondents were <30 years old, and 49.10% (220) were aged 30 years or over. The participants were distributed as 64.10% in Rabigh city and 35.90% in the surrounding villages. All the participants were educated, with most of them (60.70%) holding a bachelor's degree. The respondents comprised 37.30% students, 30.10% unemployed and the rest were employed in various sectors.

**Table 1:** Demographic aspects of the study population.

**Variable**		**Count (*n*)**	**Percentage (%)**
		***n* = 448**	
Gender	Male	118	26.30
	Female	330	73.70
Age	<30	228	50.90
	≥30	220	49.10
Region	Rabigh city	287	64.10
	Near Rabigh city	161	35.90
Education	Less than high school	13	2.90
	High school	105	23.40
	Diploma	31	6.90
	Bachelor	272	60.70
	Master	18	4.00
	PhD	9	2.00
Occupation	Student	167	37.30
	Education	41	9.20
	Not education	105	23.40
	Unemployed	135	30.10

### Adherence to COVID-19 Prevention Regulations and Awareness of the Main Symptoms and Prevention Methods Announced by the Saudi Ministry of Health

Table 2 lists the responses (from both questionnaires, that is, before and after the quarantine) to the questions that evaluated the participants' adherence to COVID-19 prevention regulations distributed by the Saudi Ministry of Health. For the questions about following the rules to prevent infection, such as wearing a face mask, handwashing, and keeping social distancing, both groups (before and after the quarantine) provided a 67.70 and 75.90% positive response, respectively. A significant difference (*p* < 0.0001) was recorded between the responding groups when it came to negative responses to these questions, indicating that after quarantine, only 0.40% of the population were not following the existing regulations. There was a significant increase in the number of hand washings per day (*p* < 0.0054), and after the quarantine, the majority of the participants (60.90%) were washing their hands (excluding prayer ablutions) between 4 and 10 times per day. Adherence to recommended handwashing hygiene was significant (*p* < 0.0001), with the greatest percentage recorded after the quarantine. The main symptoms of COVID-19 are listed in Table 3, along with the participants' *yes, no*, or *I don't know* responses. The level of knowledge was high in both groups. The main COVID-19 prevention methods announced by the Saudi Ministry of Health are listed in Table 3. Both groups responded positively to having knowledge and awareness regarding the key methods for the prevention of the spread of COVID-19, except for avoiding touching the eyes and nose, especially when outside the house, as the majority responded negatively to this section both before and after the quarantine: 93.30 and 100%, respectively.

**Table 2:** Adherence to COVID-19 prevention regulations distributed by the Saudi Ministry of Health announcements.

**Item**		**Before quarantine**	**After quarantine**	***p*-value**
		***n* = 195**	***n* = 253**	
		***n* %**	***n* %**	
Are you following the rules to prevent infection, such as wearing a face mask, washing your hands, and maintaining social distancing?	Yes No Sometimes	67.70 26.20 6.20%	75.90 0.40 23.70	=0.054 <0.0001 <0.0001
Number of times you washed your hands per day excluding prayer ablutions.	<3 From 4 to 10 More than 10	52.30 47.70 0.00	29.20 60.90 9.90	<0.0001 <0.0054 <0.0001
Are you washing your hands following Saudi Ministry of Health hand hygiene recommendations?	Yes No Sometimes	67.70 31.80 0.50	90.50 9.50 0.00	<0.0001 <0.0001 <0.2607

**Table 3:** Awareness of the main symptoms of COVID-19 and prevention methods distributed by the Saudi Ministry of Health.

	**Item**		**Before quarantine**	**After quarantine**
			***n* = 195**	***n* = 253**
			***n* %**	***n* %**
Main COVID-19 symptoms	High temperature	Yes	92.30	97.20
		No	4.60	2.80
		I don't know	3.10	0.00
	Coughing	Yes	87.20	78.70
		No	5.60	21.30
		I don't know	7.20	0.00
	Difficulty breathing	Yes	93.80	95.30
		No	2.60	4.70
		I don't know	3.60	0.00
	Headache	Yes	67.70	90.10
		No	13.30	9.90
		I don't know	19.00	0.00
	Sore throat	Yes	67.70	83.00
		No	10.30	17.00
		I don't know	22.10	0.00
	Blocked nose	Yes	56.90	59.30
		No	16.40	40.70
		I don't know	26.70	0.00
COVID-19 prevention methods	Handwashing	Yes	100.00	100.00
		No	0.00	0.00
		I don't know	0.00	0.00
	Using tissues when coughing or sneezing	Yes	94.40	98.80
		No	3.60	1.20
		I don't know	2.10	0.00
	Avoid touching the eyes and nose, especially when outside the house	Yes	0.00	0.00
		No	93.30	100.00
		I don't know	6.70	0.00
	Avoid contact with any individual outside the household	Yes	97.40	95.70
		No	1.00	4.30
		I don't know	1.50	0.00
	Avoid sharing personal items with any individual outside the household or who may be carrying COVID-19	Yes	96.90	100.00
		No	2.10	0.00
		I don't know	1.00	0.00
	Wear a mask in public places	Yes	96.40	97.60
		No	2.10	2.40
		I don't know	1.50	0.00

### Respondents' Commitment to Social Distancing Recommendations, Engagement With COVID-19 Smartphone Applications, and Sources of Information Used by the Participants During the Quarantine

**Figure 1:**
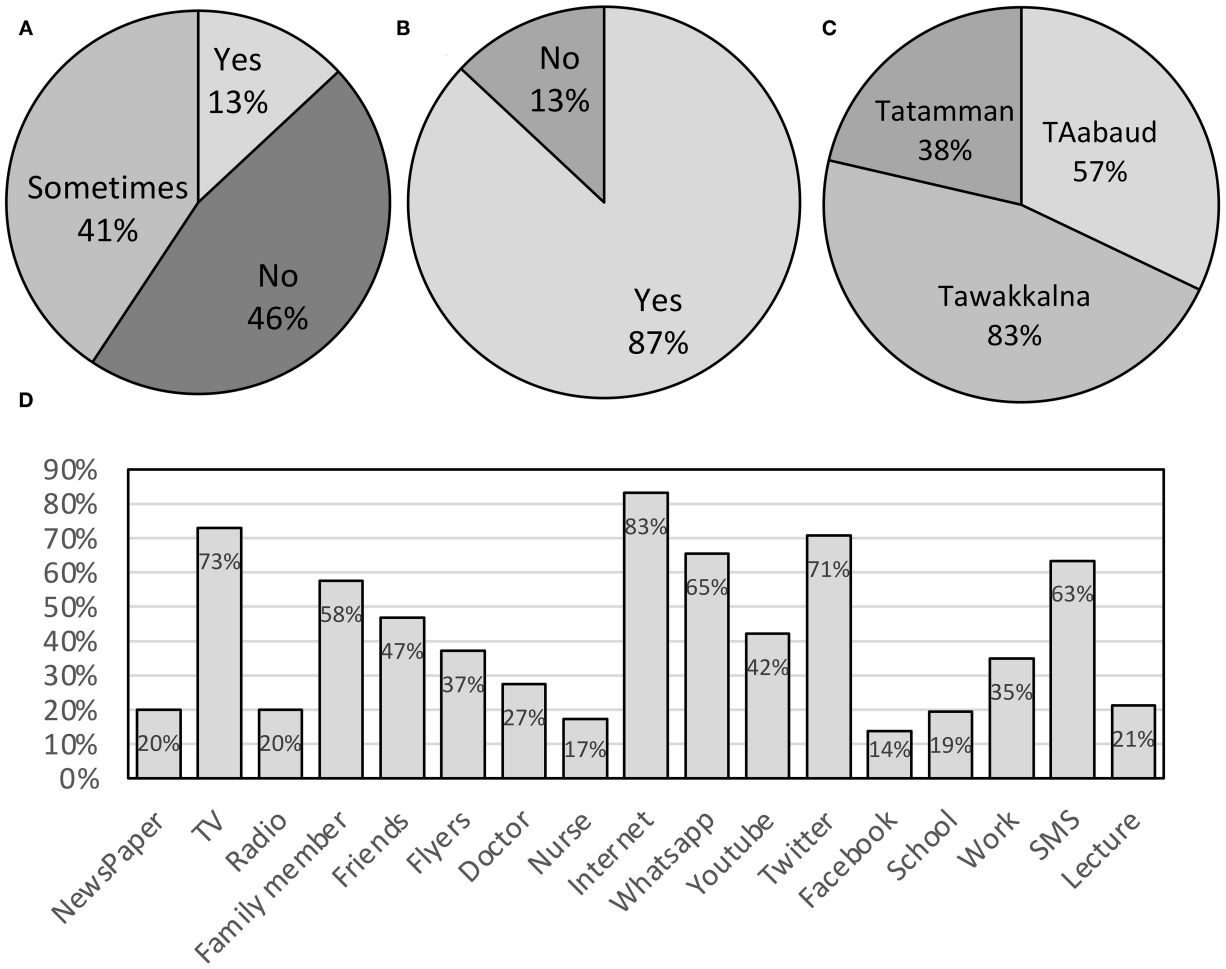
The charts present the following statistics. **(A)** Percentage of responses to the question: Did you go outside the house and not follow social distancing recommendations during the COVID-19 quarantine? Of the respondents, 46% sometimes followed the social distancing recommendations during the quarantine. **(B)** Percentages of responses to the question about whether the respondents used any COVID-19 mobile applications during the quarantine. Of the respondents, 87% used some COVID-19 mobile applications during the quarantine. **(C)** Percentages of responses to the question about the COVID-19 mobile applications the respondents used during the quarantine. The majority of the participants (47%) used Tawakkalna. **(D)** Percentages of responses to the question about where the participants obtained their information regarding symptoms, prevention and general information about COVID-19. Of the respondents, 83% primarily relied on the internet as their main source of information.

The pie chart in Figure 1A presents the percentages of the groups' responses to the question: Did you go outside the house and not follow social distancing recommendations during the COVID-19 quarantine and lockdown? This question was only applied to the group of participants who took the survey after the quarantine. The majority of this group (46%) sometimes followed the social distancing recommendation during the quarantine and 41% positively followed and were committed at all times to the recommendation, with 13% giving a negative response to the same question.

The pie chart in Figure 1B presents the percentages of the groups' responses to the question of whether they used any COVID-19 mobile applications during the quarantine, and the pie chart in Figure 1C shows the applications they used. These questions were only applicable to the group of participants who took the survey after the quarantine. During the quarantine, 87% of the participants used some COVID-19 mobile applications: the majority (47%) of the participants used Tawakkalna, whereas 32 and 21% used Tabaud and Tatamman, respectively. The bar chart in Figure 1D presents the participants' answers to the question about the sources from which they obtained their information regarding COVID-19. The majority (83%) of the participants primarily relied on the internet as their main source of COVID-19 information, and TV ranked second and Twitter ranked third, with 73 and 71%, respectively. Facebook ranked last, with 14%.

## Discussion

The goal of this research was to estimate the general level of awareness and knowledge of the prevention, symptoms and transmission control of COVID-19 before and after the quarantine of residents of Rabigh city and its adjacent villages in Saudi Arabia. Public participation is important and the most effective approach for controlling the spread of coronavirus. However, considering its novel nature, it is important to increase public consciousness when attempting to take preventive steps. To the best of our knowledge, this is the first time a questionnaire survey has been conducted to assess awareness of the COVID-19 outbreak before and after quarantine in Rabigh city, Saudi Arabia, based on crowdsourced data.

At the time of this study, during the current COVID-19 pandemic crisis, no authorized antiviral agents, medications or vaccines were available for defense against this sometimes fatal disease. However, successful prevention methods were available to limit the spread of the virus, such as wearing a face mask, practicing appropriate handwashing procedures and maintaining social distances (27). In an effort to raise awareness and push back against its spread, the Saudi Ministry of Health published guidelines and facts about COVID-19, including its sources, symptoms and methods of prevention (28), and King Abdulaziz University in Rabigh held educational courses on COVID-19 (29). In this study, females formed the largest participants in both stages. Awareness in females was better compared to males. We hypothesized that greater female representation was the explanation for the gender differences in the response rates. Although the authors attempted to distribute the sample across the target audiences in a similar manner, evidence showed that gender was influenced by online survey response activity. It was noticeable that during the first stage, the participants' awareness of COVID-19 symptoms was not very high, while during the second stage, awareness of symptoms increased by 6%. Particularly, the participants' awareness of headache and shortness of breath was increased by 23 and 2%, respectively. The participants showed good knowledge regarding hand hygiene, time required for hand hygiene and precautions overall.

Data from this study revealed that the Saudi Ministry of Health's daily report built a secure relationship and strong communication between the government and the people. The majority (46%) sometimes followed social distancing recommendations during the quarantine, and there was a significant increase in the number of hand washings per day (*p* < 0.0054). After the quarantine, the majority of the participants (60.90%) washed their hands (excluding prayer ablutions) between 4 and 10 times per day. In contrast, another survey of 385 participants from 23 countries recently revealed a lack of awareness among dentists on the key aspects of disinfection protocols during the COVID-19 pandemic due to a lack of adequate knowledge of the implementation of guidelines on disinfection, especially against COVID-19 (30). This reinforces the importance of raising awareness among the public and health professionals across various health education networks. A recent survey of 1,767 participants was conducted to investigate awareness, knowledge, and behaviors toward COVID-19 among residents of Riyadh City. Of all the participants, 95% presented a positive attitude toward practices regarding COVID-19 (31). This supports our findings regarding the successful effort of the Saudi Ministry of Health in raising public awareness to control the spread of COVID-19. Our study's key drawback is that these results are limited to Rabigh city and its adjacent villages, and a larger survey is required to reflect the complete picture of this situation.

## Conclusion

We concluded from the results of our questionnaires that the study population had remarkable knowledge about COVID-19, including etiology, symptoms, and protection, with a noticeable difference in awareness levels before and after the quarantine. This reflects the Ministry of Health's successful efforts to distribute information. However, more exhortation and information must still be provided by the Saudi Ministry of Health to curb transmission, particularly during epidemics.

## Data Availability Statement

The datasets presented in this study can be found in online repositories. The names of the repository/repositories and accession number(s) can be found in the article/Supplementary Material.

## Ethics Statement

Ethical review and approval was not required for the study on human participants in accordance with the local legislation and institutional requirements. The patients/participants provided their written informed consent to participate in this study.

## Author Contributions

MA and RA were both responsible for the conceptualization, methodology, writing of the original draft, final editing, supervision, data collection, feedback, software, validation, formal analysis, visualization and investigation, data collection, calculations, writing, reviewing, editing of the manuscript, formal analysis, preparation of the Google form, and the distribution of the survey. All authors contributed to the article and approved the submitted version.

## Conflict of Interest

The authors declare that the research was conducted in the absence of any commercial or financial relationships that could be construed as a potential conflict of interest.
