# Supplementary material for: Awareness of COVID-19 Before and After Quarantine Based on Crowdsourced Data From Rabigh City, Saudi Arabia: A Cross-Sectional and Comparative Study
Source: Front Public Health. 2021 Apr 7;9:632024. doi: 10.3389/fpubh.2021.632024 (PMC8058207; doi:10.3389/fpubh.2021.632024)
Supplement: Supplementary file 1 [file Data_Sheet_1.PDF]

Supplementary Material

1 Supplementary Data

1- Research questionnaires links:

Questionnaire's link before covid-19 compulsory quarantine and lockdown
https://docs.google.com/forms/d/e/1FAIpQLScbCjvBvOl37TExz0ArrOpPMFjCCArwKUv-i7JKYZGktuilYw/viewform?usp=sf_link

Questionnaire's linkcovid-19 compulsory quarantine and lockdown:
https://docs.google.com/forms/d/e/1FAIpQLSeqHHkrP3XPOcW-XwE1TbNuL4B-9BvW4Jv29RQC8Kd6Kr7Zow/viewform?usp=sf_link

2- Information about COVID-19 applications listed from the app store:

	**Covid-19 Application**	**App Information**	**Google Play link**	**App store Link**
**1**	Tawakkalna	Tawakkalna (Covid-19 KSA) is the official app. approved by the Saudi Ministry of Health to prevent the spread of coronavirus, and was developed by the National Information Center. The app. provides instant and live information about the number of coronavirus infections in the Kingdom, and helps in the early detection of possible infections once users show coronavirus symptoms. It allows citizens and residents to request movement permits in cases of necessity during curfew; follow-up on their permit request status during curfew; and notify them when they are close to infectious or isolated areas. Through the app., they can also report COVID-19 suspected cases to help individuals receive the health care they, or others, need.	**https://play.google.com/store/apps/details?id=sa.gov.nic.tawakkalna&hl=en_GB&gl=US**	**https://apps.apple.com/sa/app/tawakkalna-covid-19-ksa/id1506236754**
**2**	Tatamman	Tetamman is an application under the Ministry of Health in the Kingdom of Saudi Arabia, which aims to reinforce the commitment of all persons directed to isolation, and follow-up their cases. The app provides many different services, for example, to book an appointment for the Covid-19 test. Services provided in the application:<br><br>1. COVID-19 test results.<br><br>2. Direct contact with 937 to ask for help.	**https://play.google.com/store/apps/details?id=com.tetaman.home&hl=en_GB&gl=US**	**https://apps.apple.com/sa/app/tetamman-%D8%AA%D8%B7%D9%85%D9%86/id1503939198**

		3. Daily symptoms check-up.<br><br>4. update the data of who has contacted a positive case.<br><br>5. Educational Content Library.<br><br>6. Countdown indicator for isolation days.<br><br>7. Alerts through notifications, text messages, and automated calls.<br><br>The application is available in Arabic and English.		
**3**	Tabaud	Tabaud app for smartphones is one of the latest government efforts in Saudi Arabia to combat and contain the coronavirus (COVID-19) pandemic via Apple/Google Exposure Notification API. The app was developed by the National Information Center NIC of the Saudi Data and Artificial Intelligence Authority SDAIA, in close cooperation with the Ministry of Health MoH as the official contact tracing application in Saudi Arabia. The app provides 3 main services for users: notifying people if they had contact with others confirmed to be infected with coronavirus; also, providing them help by sending their health forms to the Ministry of Health to provide them necessary medical support according to the status and progress of the case; in addition to enabling those confirmed to be infected with coronavirus to voluntarily share their tests' results with people they had contact with during the past 14 days. Tabaud completely respects user privacy. Use of the app requires no information or location sharing, as it relies on Bluetooth technology to gather and refresh IDs randomly. In case the user wants to start the medical procedure of testing for coronavirus, the Ministry of Health regulations require providing main information, such as: name, National or Residence ID number, and date of birth.	**https://play.google.com/store/apps/details?id=sa.gov.nic.tabaud&hl=en_GB&gl=US**	**https://apps.apple.com/sa/app/tabaud-covid-19-ksa/id1514704802**
